# Synthesis and Evaluation of Molecularly Imprinted Silica Gel for 2-Hydroxybenzoic Acid in Aqueous Solution

**DOI:** 10.3390/ijms14035952

**Published:** 2013-03-14

**Authors:** Siti Farhana Abdul Raof, Sharifah Mohamad, Mhd Radzi Abas

**Affiliations:** Department of Chemistry, Faculty of Science, University of Malaya, Kuala Lumpur 50603, Malaysia; E-Mails: sharifahm@um.edu.my (S.M.); radzi@um.edu.my (M.R.A.)

**Keywords:** molecularly imprinted silica gel, 2-Hydroxybenzoic acid, functionalized silica gel

## Abstract

A molecularly imprinted silica gel sorbent for selective removal of 2-Hydroxybenzoic acid (2-HA) was prepared by a surface imprinting technique with a sol-gel process. The 2-HA molecularly imprinted silica gel (2-HA-MISG) sorbent was evaluated by various parameters, including the influence of pH, static, kinetic adsorption and selectivity experiments. The optimum adsorption capacity to the 2-HA appeared to be around pH 2 by the polymer. Morevoer, the imprinted sorbent displayed fast uptake kinetics, obtained within 20 min. The adsorption capacity of the 2-HA-MISG (76.2 mg g^−1^) was higher than that of the non-imprinted silica gel (NISG) (42.58 mg g^−1^). This indicates that the 2-HA-MISG offers a higher affinity for 2-HA than the NISG. The polymer displays good selectivity and exhibits good reusability. Experimental results show the potential of molecularly imprinted silica sorbent for selective removal of 2-HA.

## 1. Introduction

2-Hydroxybenzoic acid (2-HA), also known as salicylic acid, is a colorless acicular crystal and is an organic micro-molecular compound. It is widely used in many pharmaceutical and cosmetic formulations, as it is easily produced from hydrolytic deacetylation of the common drug acetylsalicylic acid (aspirin) [1]. 2-HA can act as a cosmetic product in low concentrations, but can cause serious environmental problems at high concentrations [2]. This aromatic organic compound may be harmful to consumers due to its tendency to induce allergic contact dermatitis [3]. Therefore, the efficient removal and determination of 2-HA from aqueous solutions have received considerable attention.

Molecularly imprinted polymers (MIPs) are synthetic materials which can selectively recognize a target molecule or related analogous compounds [4]. MIP preparation can be achieved by polymerization in an organic or aquatic phase, as reported by Kyzas *et al.* and Piletska *et al.*[5,6]. In a common preparation, monomers form a complex with a template and then are joined by using cross-linker agents. Removal of the template by extraction will leave the binding site which is complementary to the target analyte in size, shape and functionality [7].

MIPs have been widely used as separation media in liquid chromatography [8], sensors [9], catalyses [10] and screening [11]. MIPs have also been applied in the environmental field for ions [12,13], dyes [14,15], herbicides [16] and phenol [17], which illustrate its multi-functionality.

Studies on MIPs for 2-HA as a template have been well documented over the past years by bulk polymerization and emulsion polymerization [18–20]. Most of these materials exhibit high affinity and selectivity [21], but they possess low binding capacity and poor site accessibility to target analytes [22]. Therefore, the surface molecularly imprinted with sol-gel materials (MISGMs) have been introduced in order to overcome the shortcomings of traditional methods [23]. Recently, MISGMs have been extensively studied due to their ease of preparation [24–26].

To the best of our knowledge, there is as of yet no study reported on MISGMs for 2-HA. Herein, we report on a highly selective imprinted silica gel sorbent with binding sites situated at the surface for selective removal and separation of 2-HA. Basically, two types of sorbent have been established which are 2-HA molecularly imprinted silica gel (2-HA-MISG) and non-imprinted silica gel (NISG) to use further in the batch binding experiments. The 2-HA imprinted silica gel sorbent is characterized by the Fourier Transform Infrared Spectrometer (FTIRs), Brunauer-Emmett-Teller (BET) analysis and Scanning Electron Microscsopy (SEM) analysis. The adsorption was evaluated by studying the effect of pH, shaking time, and concentration of 2-HA. Selectivity and reusability studies also need to be taken into account, as they can affect the removal of 2-HA in aqueous solution. A validation method was established for the determination of 2-HA in a cosmetics sample.

## 2. Results and Discussion

### 2.1. Preparation of 2-HA Molecularly Imprinted Silica Gel Sorbent (2-HA-MISG)

**Figure 1:**
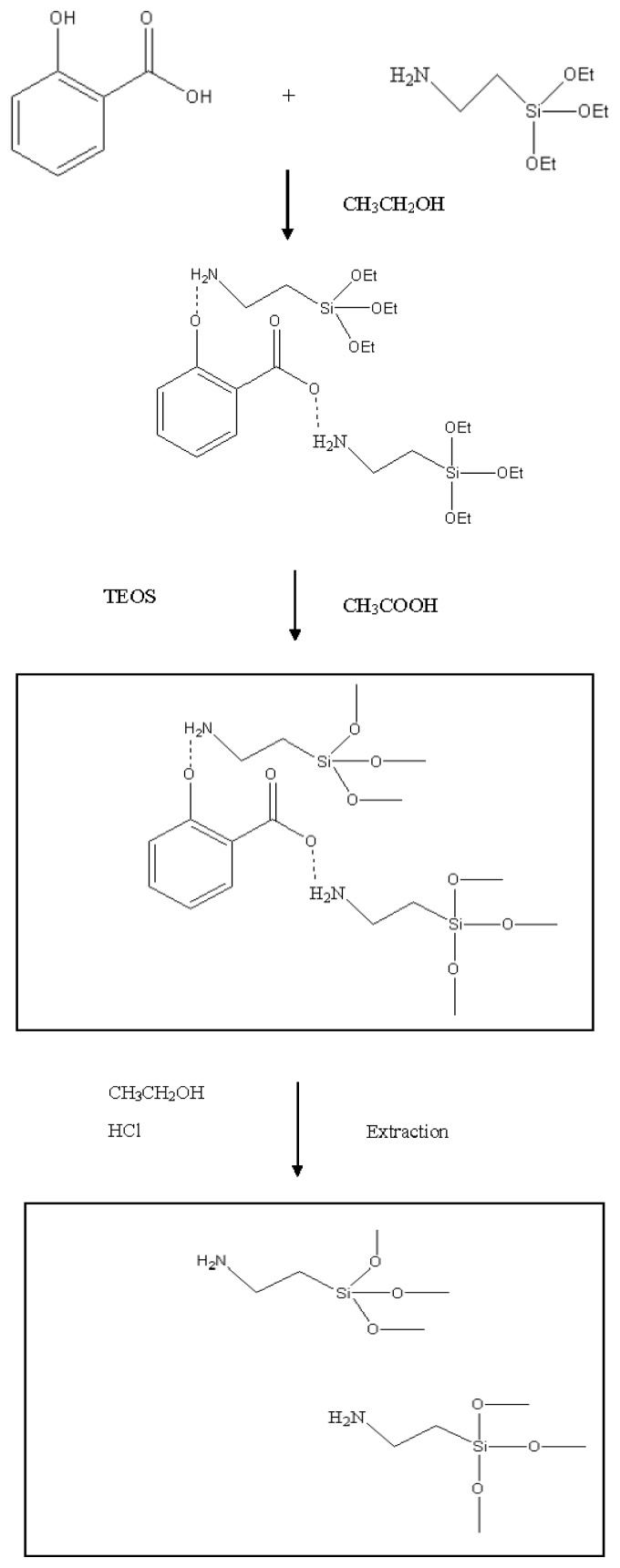
Schematic mechanism of 2-HA-MISG preparation.

The principle of molecular imprinting lies in the preservation of the pre-polymerized host/guest structure into a polymeric matrix. It is thus important that the template and the functional monomer form stable complexes through hydrogen bonding, ionic bonding, or other interaction forces in the pre-polymerization mixture [27]. Figure 1 shows the possible reaction mechanism of the 2-HA-MISG preparation. In this procedure, APTES was used as functional monomer and combined with 2-HA mainly by carboxyl groups and hydroxyl groups. The complexation between APTES and 2-HA would form strong hydrogen bonding between the amino group of APTES with hydroxyl and carboxyl groups of 2-HA [26]. TEOS acts as a cross-linker agent to maintain the stability of the recognition sites [28] under the existence of HAc catalyst [29]. HAc is often used as an activator to accelerate the formation of the polymer [30]. Meanwhile, HCl was used to interfere with the 2-HA/APTES bond for the removal of 2-HA in the polymer [25].

### 2.2. Characterization

#### Physical Properties

**Figure 2:**
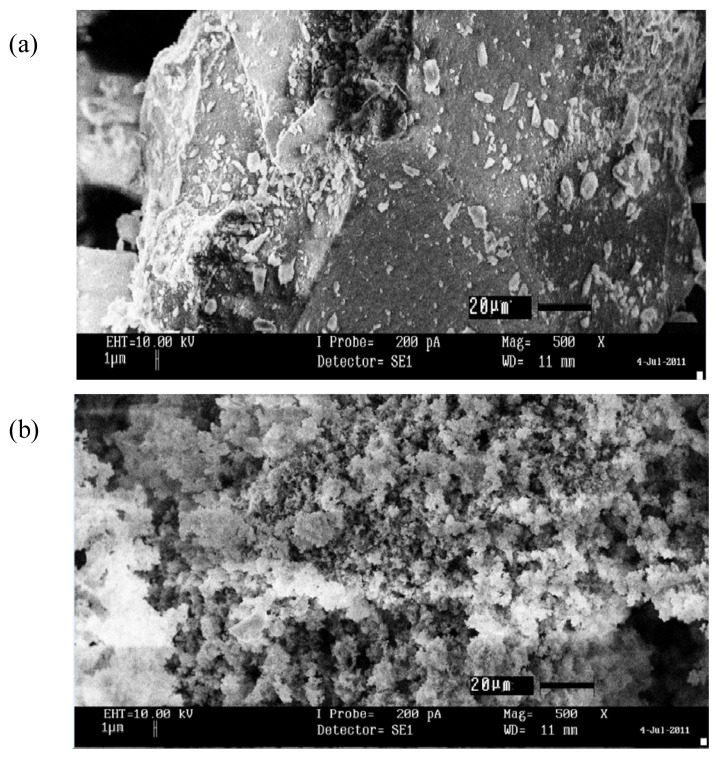
SEM micrographs of (**a**) 2-HA-MISG (×500 magnification) and (**b**) NISG (×500 magnification).

The morphology of 2-HA-MISG and NISG is shown in Figure 2. For both polymers, it can be seen that the agglomerates of microparticles come in different sizes. The particles possess a porous surface due to pores with irregular shapes and sizes, which can play an important role in the adsorption process. The roughness of the particle surface itself causes the increase in the surface area [31]. However, the particles of NISG possess a uniform, compact and smooth shape which have an irregular and rough morphology [31]. The structure, due to the lack of specific binding sites, has been created for 2-HA-MISG.

Nitrogen adsorption/desorption analysis of Brunauer-Emmett-Teller (BET) was used to evaluate the BET surface area, pore volume and pore size of the sorbent and this is shown in Table 1.

It is proven that 2-HA-MISG (123.4 m^2^g^−1^) has a larger specific area than NISG (102.2 m^2^g^−1^). This is due to the imprinting of the template (2-HA) on the surface of the 2-HA-MISG sorbent, thereby resulting in particles having larger surface area [31]. These data support the observation from the SEM images.

**Figure 3:**
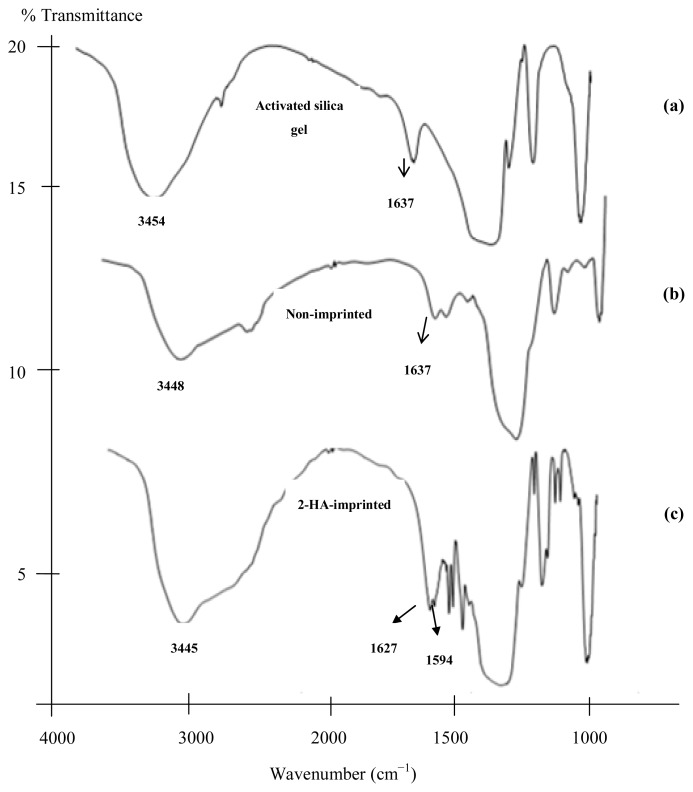
IR spectra of activated silica gel (**a**), 2-HA-imprinted (**b**) and non-imprinted sorbents (**c**).

The IR employed for activated silica gel, 2-HA-imprinted, and non-imprinted silica gel sorbents are compared in Figure 3. The IR spectrum of activated SiO_2_ shows that the binding bands around 1096.31 cm^−1^ and 974.62 cm^−1^ indicate that there are Si–O–Si vibration and Si–O–H stretching vibrations, respectively. Similarly, the peaks around 803.66 cm^−1^ and 467.22 cm^−1^, respectively were attributed to Si–O vibrations in the form of silica particles. Imprinted and non-imprinted sorbents show similar locations and appearances of the major bands. The absorption at 1627 cm^−1^ and 1637 cm^−1^ for 2-HA-MISG and NISG, respectively, can be assigned to the C=O group. The common characteristics of the imprinted and non-imprinted sorbents when compared with activated silica gel were the N–H bond around 1594.40 cm^−1^, and a C=O bond around 1627 cm^−1^ (Figure 3). These results further indicate that −NH_2_ had been grafted onto the surface of activated silica gel after modification, so the APTES had been combined with the surface of the functionalized silica gel sorbent.

### 2.3. Evaluation of Adsorption

#### 2.3.1. Effect of pH

**Figure 4:**
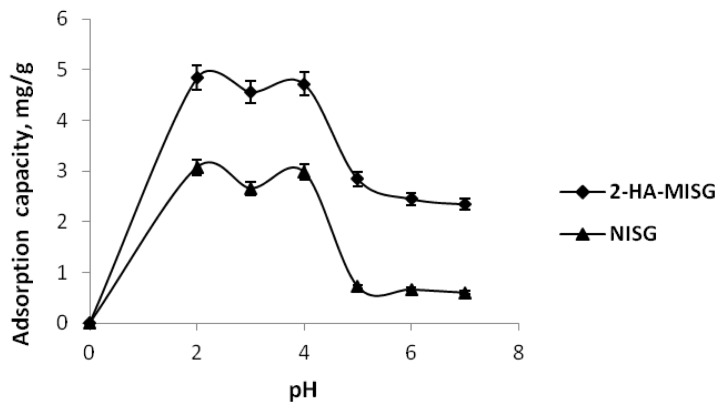
Effect of pH on 2-HA adsorption.

pH is a significant factor for the adsorption process due to its properties of imprinted surface and the speciation of the target compound. Therefore, it is necessary to study the effect of pH on adsorption [32]. Figure 4 shows the effect of pH on the 2-HA adsorption by 2-HA-MISG and NISG. It can be seen that the adsorption capacity of the 2-HA appeared between pH 2 to pH 4 by the polymers. The adsorption process slightly increases, but it was significantly decreasing from pH 5 to pH 7.

This could be interpreted as the ionization of 2-HA. 2-HA at low pH is almost undissociated (weak acid, pKa = 2.98). Therefore, controlling the pH of the solution to match the pKa can cause the 2-HA to change its deprotonated or protonated condition. The relative ability for a molecule to give up a proton (deprotonate) is measured by its pKa value. In addition, if the pH values are higher than the pKa values of 2-HA, this would result in a mostly deprotonated compound which leads to the decrease in removal efficiency. Under these conditions, the imprinted surfaces are generally covered with amino groups in various forms at different pH levels [33]. As pH is becoming lower (pH 2, pH 3 and pH 4), amino groups were protonated to form the positive sites (for example −NH_3_^+^ groups) and the electrostatic attraction occurred between the 2-HA analyte solution and −NH_3_^+^, leading to increased removal efficiency under strong acidic conditions. Meanwhile, at higher pH values, the concentration of H^+^ had decreased and, at the same time, the concentration of OH^−^ increased, which competed with the 2-HA analyte solution. Therefore, the ability of −NH_2_ to be protonated had weakened, thus resulting in the decline in removal efficiency [33]. Therefore, pH 2 was chosen due to the maximum adsorption capacity for the following adsorption test. It was also found that the 2-HA-MISG had higher sorption efficiency than the NISG over the entire pH range investigated, showing a good imprinting effect and adsorption performance.

#### 2.3.2. Effect of Time

**Figure 5:**
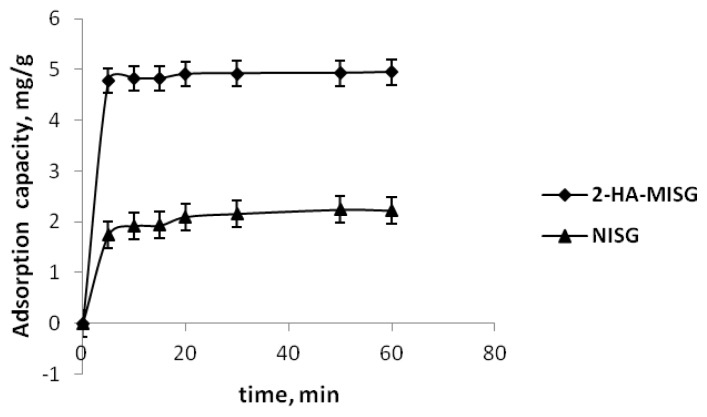
Effect of time on 2-HA adsorption.

The kinetic profile of 2-HA-MISG and NISG was examined at a 2-HA concentration of 10 ppm and this is illustrated in Figure 5. It was very apparent that a significant 2-HA-adsorption equilibrium on 2-HA-MISG and NISG occurred in 20 min and no appreciable changes had been observed in terms of adsorption after that. It was a much higher adsorption capacity for the 2-HA binding onto the MISG sorbent compared with the NISG. This indicates that a molecular imprinting process had resulted in the formation of specific recognition sites on the surface of the 2-HA-MISG sorbent.

#### 2.3.3. Effect of Concentration

**Figure 6:**
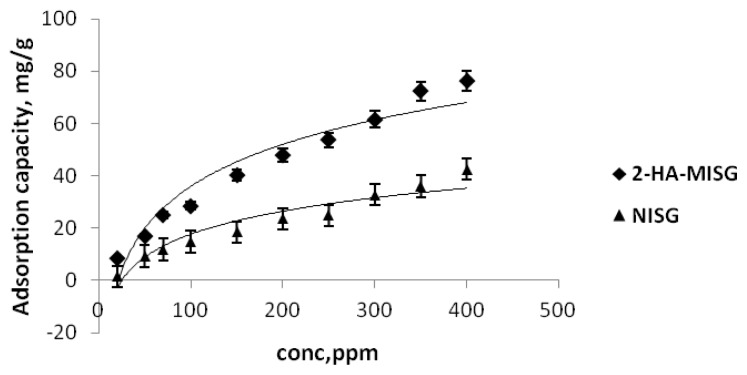
Effect of concentration on 2-HA adsorption.

Figure 6 shows the adsorption isotherm of 2-HA onto MISG and NISG sorbents. It can be noted that the adsorption capacities of 2-HA-MISG and NISG sorbents had increased with the initial increase of the concentrations of 2-HA. The adsorption capacities of the 2-HA-MISG and NISG were calculated as 76.2 mg g^−1^ and 42.58 mg g^−1^ respectively. The adsorption capacity of the 2-HA imprinted sorbent was about 1.8 times higher than that of the non-imprinted sorbent. It shows that the 2-HA-imprinted sorbent had had a much higher affinity for 2-HA than the non-imprinted counterpart.

#### 2.3.4. Binding Selectivity of the Sorbent

**Figure 7:**
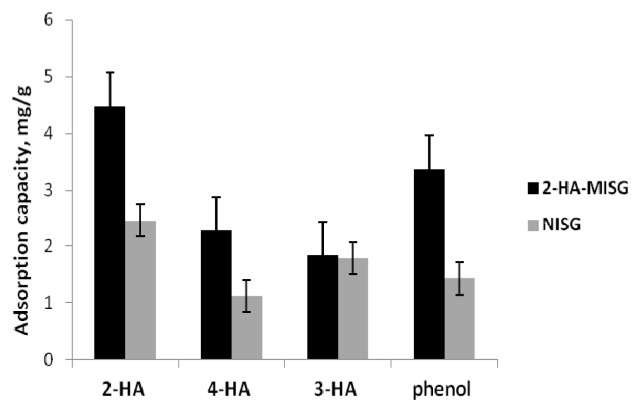
Selectivity study on 2-HA, 3-HA, 4-HA, and phenol.

Adsorption and competitive recognition studies were performed with 2-HA, and they are the structurally related compounds of 3-HA, 4-HA, and phenol, as shown in Figure 7.

Table 2 summarizes the data for adsorption capacity, distribution coefficient (K), selectivity coefficient of the sorbent (α) and the imprinting factor (IF) obtained in these competitive binding experiments. The α values for the imprinted sorbent with the corresponding non-imprinted sorbent have indicated that there is a significant increase in α for 2-HA through imprinting [34]. For example, the α (2-HA/3-HA) value of the imprinted sorbent (14.59) is 8.3-fold that of the non-imprinted sorbent (1.9). The large α value of the imprinted sorbent is indicative of its high selectivity for 2-HA over the related compounds. This might result from the imprinting effect [35] and acidity [34]. The result supports the previous study by Li *et al.*[20] in which 2-HA-MIPs show higher binding selectivity compared to 4-HA and sulfosalicylic acid. 2-HA exhibited higher acidity (pKa 2.98) compared to 3-HA (pKa = 4.08), 4-HA (pKa = 4.54) and phenol (pKa = 9.95) due to the fact that it is easily deprotonated [36]. This can be explained by the condition whereby the structure of deprotonated 2-HA is very stable due to the formation of a double ring structure by the intramolecular hydrogen bond which can stabilize the conjugate base anion by resonance effect [36]. Hence, the interaction of 2-HA with the −NH_2_ group is stronger than that of the 3-HA, 4-HA and phenol.

### 2.4. Reusability

The imprinted sorbent was used to extract 2-HA through five extraction cycles. The mixture solution of ethanol and HCl was used to strip the adsorbed 2-HA, before the material was filtered and neutralized with 0.1 M NaOH, and washed with deionized water. The results are shown in Table 3. This result suggests that the sorbent can be used repeatedly and the recognition sites are stable [26].

### 2.5. Application to Real Samples

To validate the developed method, the recoveries of 2-HA at different spiking levels for the cosmetic samples and the results are listed in Table 4. The results have pointed out that the recoveries of 2-HA were in the range from 86.89% to 105%. Relative standard deviation (RSD) values ranged from 0.05% to 0.97% in all cases. All the results indicate that the developed method has satisfactory accuracy and is practical for the determination of 2-HA in cosmetic samples.

## 3. Experimental Section

### 3.1. Chemicals

Silica gel (60–200 mesh, Sigma Aldrich, St. Louis, Missouri, MO, USA) was used as the support to prepare 2-HA-imprinted functionalized sorbent. Tetraethoxysilane (TEOS), 3-Aminopropyltriethoxysilane (APTES) (Sigma Aldrich, St. Louis, Missouri, MO, USA), 2-HA (Merck, Darmstadt, Germany), 3-Hydroxybenzoic acid (3-HA) (Merck, Darmstadt, Germany), 4-Hydroxybenzoic acid (4-HA) (Merck, Darmstadt, Germany), Acetic acid (HAc) (Sigma Aldrich, St. Louis, Missouri, MO, USA) and Natrium hydroxide (NaOH) (R and M chemicals) were used in this study.

### 3.2. Instrumentation

The HPLC-UV analysis was performed using a chromatographic system, a CBM-20A communication bus module from Shimadzu, Japan. A HPLC-UV system consists of a LC-20AT pump, a SPD-M20A diode array detector, a SIL-20AHT auto sampler and a CTO-20AC column oven. All separations were achieved on an analytically reversed-phase Chromolith RP-18 monolithic column (100 mm × 4.6 mm i.d., Merck, Germany) under isocratic conditions at a column temperature 35 °C with a mobile phase containing acetonitrile/acid in water (50:50, *v*/*v*) which is formic acid (0.1 M), at a flow rate of 0.8 mL min^−1^.

### 3.3. Characterization of the 2-HA Imprinted Silica Gel Sorbent

The IR spectra were obtained by using the Perkin-Elmer RX1 FT-IR spectrometer with samples prepared as KBr pellets. All the spectra were run in the range of 400–4000 cm^−1^ at room temperature. The IR was carried out for MISG, NISG, and activated silica gel.

The Brunauer-Emmett-Teller (BET) analysis was carried out to determine the specific area and average pore diameter of a material. The analysis was conducted by performing nitrogen adsorption at liquid nitrogen at temperature (77 K). Typically, at least 1 g of sample (MISG and NISG) was used each time. The sample was degassed overnight. The specific surface area was calculated using the BET method.

Samples (MISG and NISG) had been evaluated by a scanning electron microscope (Model LEO 1450 VPSEM, United Kingdom) using the scanning electron microscopy (SEM) in order to evaluate the morphology and surface structure of the sample itself. The samples were attached to a stub using a double-sided adhesive tape. Meanwhile, the samples were coated with gold and examined at 20 kV.

### 3.4. Preparation of 2-HA-Imprinted Silica Gel Sorbent

Silica gel was heated overnight to activate the surface. As proposed by Han [34], 1 g of 2-HA was dissolved in 5 mL of ethanol, and mixed with 2 mL of APTES to prepare the sorbent. The mixture was stirred for 20 min, and then 4 mL of TEOS was added. After stirring for 5 min, 1 g of activated silica gel and 1 mL of 1 M HAc (as catalyst) were added. The mixture began to co-hydrolyze and co-condense after stirring for a few minutes, then it was incubated for 10 h at room temperature. The product was filtrated and dried in a vacuum oven at 100 °C for 8 h. Thus, the activated silica gel surface was grafted with the complex. For comparison, the non-imprinted functionalized silica gel sorbent was also prepared using an identical procedure, but without the addition of 2-HA.

### 3.5. Extraction of Template

The sorbent was extracted with ethanol and 6 M HCl via stirring for 2 h to remove 2-HA. The product was isolated by filtration, washed with the mixture of ethanol and 1 M HCl, neutralized with 0.1 M NaOH, and then washed with pure water. This procedure was repeated several times until no template molecules were detected, or they came in very low concentration. Finally, the sorbent was dried in the oven at 80 °C for 12 h [34].

### 3.6. Adsorption Study

To test the effect of pH, 20 mg of 2-HA-MISG sorbent was equilibrated with 10 mL of 2-HA solutions containing 10 ppm with different pH values from 2 to 7. To measure the adsorption capacity, static adsorption tests were carried out. 20 mg of MISG was added to 10 mL of various concentrations of 2-HA solutions with optimum pH. Uptake kinetics of 2-HA-MISG was also examined. 2-HA-MISG sorbent was added to 10 mL of 10 ppm of 2-HA solution. The mixture was mechanically shaken for 5, 10, 15, 20, 30, 50 and 60 min at room temperature, respectively, then centrifugally separated and filtered. The free 2-HA concentration in the filtrate was detected by HPLC.

The selectivity adsorption experiments were conducted by preparing a single solution of 3-HA, 4-HA and phenol solution with each initial concentration of 10 ppm. 3-HA, 4-HA or phenol was used as the comparative agents since their chemical molecular structures are similar to 2-HA to a certain extent. The adsorption of 2-HA to NISG was also measured in a similar manner. All the experiments were performed in triplicate.

### 3.7. Application to Real Samples

One gram of the cosmetic product was accurately weighed in a 10 mL volumetric flask and diluted with ethanol and the cosmetic samples were spiked with an appropriate amount of 2-HA solution with an additional 20 mg of sorbent. The mixtures were mechanically shaken for 60 min at room temperature and then centrifugally separated. The filtrate was measured for the unextracted 2-HA by HPLC.

## 4. Conclusions

In this study, a simple molecular imprinting procedure was adopted to synthesize a highly selective 2-HA-imprinted silica gel sorbent by combining a surface molecular imprinting technique with a sol-gel process. The adsorption process was very quick for the sorbent and the adsorption equilibrium can be achieved within 20 min. The adsorption capacity of the 2-HA-MISG (76.2 mg g^−1^) was higher than that of the NISG (42.58 mg g^−1^). This implies that the MISG offered a higher affinity for 2-HA than the NISG. Moreover, 2-HA-MISG has a superior reusability and stability which can be repeatedly used in five sequential cycles with recoveries no less than 90%. Therefore, the imprinted material prepared shows advantageous characteristics such as fast adsorption and high affinity that enable it to be selected and regenerated for 2-HA. This result shows the potential of molecularly imprinted silica sorbent for the selective removal of 2-HA in cosmetic samples.

## Figures and Tables

**Table 1:** Porosities of particles determined by the BET analysis.

Sample	2-HA-MISG	NISG
BET surface area (m^2^g^−1^)	123.4	102.2
Pore volume (m^3^g^−1^)	0.4067	0.2873
Pore size (nm)	131.9	112.5

**Table 2:** Competitive loading of 2-HA, 4-HA, 3-HA and phenol by the imprinted and non-imprinted sorbents.

	Sorbents	Imprinted *	Non-imprinted *
K	2-HA	4.257	0.485
	4-HA	0.3193	0.1207
	3-HA	0.2918	0.277
	phenol	1.05	0.2013
α	4-HA	13.33	4.018
	3-HA	14.59	1.751
	phenol	4.054	2.409
IF	4-HA	3.318	-
	3-HA	8.3332	-
	phenol	1.683	-

distribution coefficient, *K* = {(*C*_i_ − *C*_f_)/*C*_f_} × {volume of solution (mL)}/{mass of sorbent (g)}, where *C*_i_ and C_f_ represent the initial and final concentrations, respectively; selectivity coefficient, α = *K*(2-HA)/*K*(4-HA or 3-HA or phenol); imprinting factor, IF = IF_imprinted_/IF_non-imprinted_; Initial concentration, *C*_i_ = 10 ppm;

* results based on three replicate analyses for all analytes.

**Table 3:** Extraction recyclability through five extraction/stripping cycle.

Extraction cycle	Loading capacity (mg/g)	Uptake (%)
1	4.123	82.88
2	4.317	86.34
3	4.335	87.13
4	4.298	86.39
5	4.354	87.07

**Table 4:** Method validation of 2-HA in real samples at different spiking levels.

Samples	Spike (ppm)	Recovery (%)	RSD (%)

		*n* = 3	*n* = 3
1	0.2	86.89	0.36
	1	93.02	0.61
2	0.2	98.27	0.05
	1	101.9	0.18
3	0.2	105	0.23
	1	96.2	0.97
4	0.2	91.2	1.60
	1	96.92	1.66
